# The effect of process variables on the physical properties and microstructure of HOPO nanoemulsion flakes obtained by refractance window

**DOI:** 10.1038/s41598-021-88381-7

**Published:** 2021-04-30

**Authors:** M. Hernández-Carrión, M. Moyano-Molano, L. Ricaurte, A. Clavijo-Romero, M. X. Quintanilla-Carvajal

**Affiliations:** Facultad de Ingeniería, Universidad de la Sabana, Km 7 vía autopista Norte, Bogotá, Colombia

**Keywords:** Oils, Chemical engineering

## Abstract

Refractance window (RW) drying is considered an emerging technique in the food field due to its scalability, energy efficiency, cost and end-product quality. It can be used for obtaining flakes from high-oleic palm oil (HOPO) nanoemulsions containing a high concentration of temperature-sensitive active compounds. This work was thus aimed at studying the effect of temperature, thickness of the film drying, nanoemulsion process conditions, and emulsion formulation on the flakes’ physical properties and microstructure. The results showed that HOPO flakes had good physical characteristics: 1.4% to 5.6% moisture content and 0.26 to 0.58 a_w_. Regarding microstructure, lower fractal dimension (FDt) was obtained when RW drying temperature increased, which is related to more regular surfaces. The results indicated that flakes with optimal physical properties can be obtained by RW drying of HOPO nanoemulsions.

## Introduction

Nanoencapsulation involves packing substances into a miniature-sized vessel and refers to bioactive packing on a nanoscale range. It is characterised by increasing encapsulated active compound bioavailability and protection against environmental and processing effects^1,2^. Nanoencapsulation often begins with the production of nanoemulsions. These are emulsions with an average droplet size of 300 nm^3^, containing oil, water and an emulsifier, which is a critical factor for creating small-sized droplets as this decreases interfacial tension (i.e. surface energy per unit area) between an emulsion’s oil and water phases^4^. Nanoemulsions used in the food science and industry fields have mainly been focused on lipid nanoparticle delivery or bioactive compound release systems due to their minimum impact on sensory characteristics and high bioavailability^5,6^.

One of the process which allows to obtain nanoemulsion is microfluidization^7^. This technique has been widely used and represents a highly efficient method for producing nanoemulsions containing small-sized droplets (100–500 nm)^8^. It uses high pressure to force fluid into specially configured microchannels^9^. Apart from inertia regarding turbulent flow, laminar elongational flow and cavitation also contribute to droplet break-up^10^.

On the other hand, Refractance Window (RW) drying is a novel drying system which converts foods into flakes or powders^11^. Here, thermal energy is transferred from hot water (95–97 °C), which is circulated beneath a plastic conveyer belt (Mylar) and used to dry a thin layer of product on the belt surface^12^. Studies have suggested that using a thin plastic belt which is transparent to infrared radiation (IR) creates a “window” for thermal energy to become transferred from hot water to wet food material^13^. As a product becomes dried as a thin film and cold air is circulated over the food layer, the heated surface is much lower (70–80 °C) compared to drum drying (120–150 °C); this means that RW-dried products typically have excellent colour, vitamin and antioxidant retention, compared to conventional drying methods^14^.

Image analysis represents a particularly useful tool for characterising food morphology. Many food materials present highly irregular structures that elude precise quantification by conventional techniques. This methodology enables measurements to be obtained from digitalised images^15,16^. Texture is an important characteristic used in identifying objects or regions of interest in an image, a grey level co-occurrence matrix (GLCM) (an image processing technique) has been widely used for measuring texture in images; image analysis can highlight textural features, such as angular second moment, contrast, correlation, inverse difference moment and entropy, which can relate to the quality state of the microstructure of the food^17,18^. Also, fractal dimension measures can be used to describe fractured surfaces quantitatively. A greater fractal dimension DF means a more tortuous fracture surface.

Regarding the formulation of microfluidization-obtained oil-in-water nanoemulsions, several authors have studied the effect of the ingredients used in them on their stability^19–22^. Other studies have focused on comparing drying technologies’ (including RW) effect on the physicochemical properties and content of functional compounds on vegetable matrices^14,23–27^. However, the effect of the process variables of RW drying on high-oleic palm oil (HOPO) nanoemulsions’ physical and microstructural properties are not reported in the literature.

The present work was thus aimed at studying the effect of process variables (drying temperature, sample thickness, microfluidization pressure) and formulation on RW-dried HOPO nanoemulsions’ physical properties (moisture, water activity, contact angle and colour) and microstructure to ensure obtaining a dried product having high oil content.

## Materials and methods

### Material

High-oleic palm oil was obtained from Fedepalma, Bogota, Colombia; whey powder was bought in a local market in Bogota, Colombia; soy lecithin was obtained from Bellchem International, Medellin, Colombia; gum Arabic powder from C. E. Roeper GmbH, Hamburg, Germany; and native corn starch from Cimpa SAS, Bogota, Colombia.

### Preparation of coarse emulsions

The coarse emulsions were homogenized in an mixer (Imusa, Bogota, Colombia), first the aqueous phase of the emulsion was prepared incorporating whey powder (formulation A: 29.76% (w/w); formulation B 29.43% (w/w)), determined according to preliminary results^28^, followed by the sequential addition of native corn starch (0.24% w/w, formulation A) or gum Arabic (0.57% w/w, formulation B). Then it was followed by the addition of HOPO (14%, w/w) to the aqueous phase and mixed over 2 min. The soy lecithin concentration was held constant at 10% w/w with respect to the HOPO concentration (1.4%, w/w) and it was added to the oil phase in the preparation of the coarse emulsion. Subsequently, emulsions were processed to obtain the nanoemulsions.

### Nanoemulsion preparation

**Figure1:**
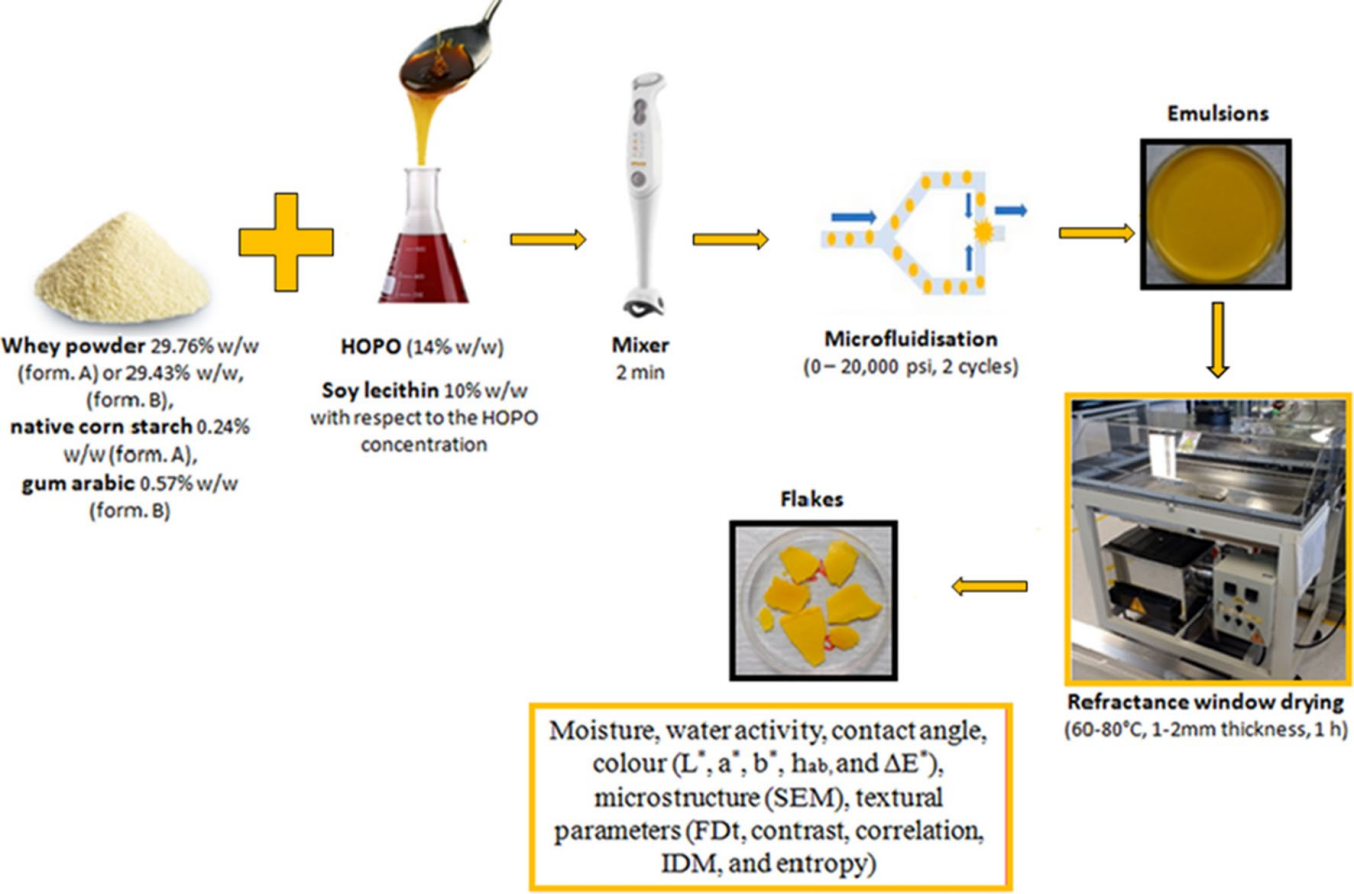
Graphical description of the process to obtain HOPO nanoemulsions and flakes.

The nanoemulsions were obtained following the methodology of Quintanilla-Carvajal et al.^29^ with some modifications. They were homogenized in an LM10 microfluidizer (Microfluidics, England) variating the operation pressure between 0 and 20,000 psi following a Box-Behnken optimization design obtained from the software Design Expert Version 10.1.0 (Stat-Ease Inc., MN, USA), in which the following two numerical factors and one categorical were also varied accordingly to their impact on the process: drying temperature (60–80 °C), sample thickness (1–2 mm), and the formulation (A and B), respectively. Table 1 shows the conditions provided by the Box-Behnken optimization design for the preparation of HOPO nanoemulsions. Figure 1 shows a graphical description of the procedure used to obtain the HOPO nanoemulsions and flakes.

**Table 1:** Box Behnken experimental design methodology for preparation of emulsions and adjusted variables to the model: moisture, water activity (aw), Contact angle (CA), Hue (h_ab_°) and change of color (ΔE*) for flakes of HOPO nanoemulsions.

Run	Factor 1	Factor 2	Factor 3	Factor 4	Moisture (%)	a_w_	CA (°)	h_ab_ (°)	ΔE*
	A: temperature (°C)	B: thickness (mm)	C: pressure (psi)	D: formulation					
1	80	1.5	20,000	A	2.03	0.3440	26.78	76.06	8.53
2	70	1.5	10,000	A	1.70	0.3711	24.94	76.72	8.60
3	80	2	10,000	A	2.24	0.3435	32.00	76.15	6.71
4	80	1.5	0	B	1.80	0.3486	26.18	73.77	11.34
5	60	2	10,000	A	3.18	0.4151	21.75	76.18	7.77
6	70	1.5	10,000	B	1.40	0.3786	22.20	75.43	10.36
7	70	1.5	10,000	B	1.80	0.3457	20.26	76.47	13.29
8	70	2	20,000	A	2.77	0.3314	20.70	77.05	7.73
9	60	1.5	20,000	A	4.56	0.3396	29.10	77.61	8.05
10	60	2	10,000	B	3.44	0.4557	19.15	76.60	7.80
11	70	1	0	A	3.42	0.3013	22.99	76.52	8.87
12	60	1	10,000	B	3.46	0.3519	23.24	77.46	10.34
13	80	1.5	20,000	B	1.79	0.3177	29.42	77.65	6.49
14	70	1.5	10,000	B	2.85	0.3052	23.69	75.76	10.98
15	60	1	10,000	A	4.84	0.5806	25.57	77.70	8.71
16	70	1.5	10,000	B	4.07	0.3255	26.65	76.96	12.62
17	70	1.5	10,000	A	3.46	0.3188	24.25	78.61	3.81
18	80	1	10,000	B	3.68	0.3628	29.95	76.64	6.04
19	70	2	20,000	B	3.79	0.3579	22.92	74.75	8.76
20	60	1.5	20,000	B	5.65	0.4452	20.45	75.67	8.16
21	70	2	0	A	2.16	0.3449	21.01	73.87	9.20
22	70	2	0	B	2.79	0.3641	20.61	73.81	9.94
23	60	1.5	0	A	4.49	0.4138	22.06	73.24	10.48
24	80	1.5	0	A	2.48	0.2872	29.73	74.27	10.38
25	70	1	20,000	A	2.42	0.2816	20.69	78.17	11.05
26	70	1.5	10,000	A	2.76	0.3310	22.73	75.79	8.24
27	70	1.5	10,000	A	3.57	0.2680	17.27	78.80	8.81
28	60	1.5	0	B	3.49	0.4590	18.88	76.73	8.50
29	70	1	0	B	2.87	0.3183	21.18	76.01	8.38
30	80	2	10,000	B	2.18	0.3070	30.57	76.39	10.20
31	70	1	20,000	B	3.33	0.3675	15.72	77.04	6.03
32	70	1.5	10,000	B	2.91	0.3642	19.42	76.77	7.66
33	70	1.5	10,000	A	4.58	0.3539	21.04	77.91	11.45
34	80	1	10,000	A	2.71	0.3323	26.25	78.67	11.91

Run	Factor 1	Factor 2	Factor 3	Factor 4	FDt	Contrast	Correlation	IDM	Entropy
	A: temperature (°C)	B: thickness (mm)	C: pressure (psi)	D: formulation					
1	80	1.5	20,000	A	2.5819	576.36	8.176 × 10^–4^	0.052	9.142
2	70	1.5	10,000	A	2.5823	562.93	7.903 × 10^–4^	0.053	9.236
3	80	2	10,000	A	2.5628	542.28	9.004 × 10^–4^	0.053	9.056
4	80	1.5	0	B	2.5884	646.24	7.343 × 10^–4^	0.049	9.299
5	60	2	10,000	A	2.6477	1011.11	4.805 × 10^–4^	0.049	9.569
6	70	1.5	10,000	B	2.5746	570.87	8.420 × 10^–4^	0.052	9.117
7	70	1.5	10,000	B	2.5661	576.00	8.605 × 10^–4^	0.051	9.044
8	70	2	20,000	A	2.5577	566.45	8.716 × 10^–4^	0.052	9.053
9	60	1.5	20,000	A	2.6390	980.59	5.054 × 10^–4^	0.039	9.528
10	60	2	10,000	B	2.6463	947.07	5.244 × 10^–4^	0.040	9.562
11	70	1	0	A	2.5763	679.40	7.206 × 10^–4^	0.047	9.297
12	60	1	10,000	B	2.6632	1042.35	4.765 × 10^–4^	0.038	9.670
13	80	1.5	20,000	B	2.5740	557.21	8.359 × 10^–4^	0.053	9.158
14	70	1.5	10,000	B	2.6055	744.08	6.711 × 10^–4^	0.045	9.295
15	60	1	10,000	A	2.6488	946.88	5.243 × 10^–4^	0.041	9.558
16	70	1.5	10,000	B	2.5835	548.90	8.318 × 10^–4^	0.053	9.102
17	70	1.5	10,000	A	2.5675	552.89	8.501 × 10^–4^	0.052	9.129
18	80	1	10,000	B	2.5886	588.15	8.071 × 10^–4^	0.051	9.175
19	70	2	20,000	B	2.5598	535.17	8.415 × 10^–4^	0.053	9.186
20	60	1.5	20,000	B	2.6388	943.09	5.314 × 10^–4^	0.040	9.129
21	70	2	0	A	2.5481	567.07	8.395 × 10^–4^	0.052	9.166
22	70	2	0	B	2.5566	554.99	7.766 × 10^–4^	0.053	9.268
23	60	1.5	0	A	2.5980	743.88	5.833 × 10^–4^	0.046	9.486
24	80	1.5	0	A	2.5578	631.66	7.84Ex10^–4^	0.049	9.189
25	70	1	20,000	A	2.5842	574.64	8.336 × 10^–4^	0.052	9.147
26	70	1.5	10,000	A	2.6073	815.98	6.028 × 10^–4^	0.043	9.290
27	70	1.5	10,000	A	2.5897	593.05	7.980 × 10^–4^	0.051	9.199
28	60	1.5	0	B	2.6202	845.60	5.012 × 10^–4^	0.042	9.710
29	70	1	0	B	2.5400	558.19	8.504 × 10^–4^	0.052	9.159
30	80	2	10,000	B	2.6010	623.01	7.503 × 10^–4^	0.050	9.270
31	70	1	20,000	B	2.5645	556.09	8.927 × 10^–4^	0.053	9.001
32	70	1.5	10,000	B	2.5524	524.56	9.457 × 10^–4^	0.054	8.978
33	70	1.5	10,000	A	2.5475	495.47	9.894 × 10^–4^	0.055	8.891
34	80	1	10,000	A	2.5988	640.00	7.444 × 10^–4^	0.049	9.255

Box Behnken optimal experimental design methodology for preparation of emulsions and adjusted variables to the model: FDt, Contrast, Correlation, IDM, and Entropy for flakes of HOPO nanoemulsions.

### Refractance window drying (RW)

A pilot scale Refractance Window (RW) dryer was used for drying nanoemulsions of HOPO. The dryer has an effective surface drying area of 0.43 m^2^ and length of 0.92 m. The main components of the dryer included a belt made of “Mylar” (polyethylene terephthalate) plastic, a water pump, a hot water tank, a heating unit, two water flumes, a suction blower, a spreader, and a scraper. The drying was accomplished by spreading HOPO nanoemulsions on the plastic belt. Molds of 14 × 24 × 0.3 cm were used to control the size of the samples. The thickness of the nanoemulsion on the belt was 1–2 mm and was controlled using a spreader bar. The thermal energy from the circulating hot water (transferred to the nanoemulsion through the belt) was used to remove moisture from the product^30^. During drying operation, the temperature of circulating hot water was maintained at 60 °C, 70 °C and 80 °C according to the experimental design. The temperature of the circulating hot water was continuously monitored at the flume inlet and outlet section using pre-calibrated Type T thermocouple sensors. Water vapor removal from the samples was facilitated by using a suction blower. The residence time to dry the HOPO nanoemulsions into flakes was set at 1 h, this time was determined according to preliminary studies (data not shown).

### Physical properties of the flakes

#### Moisture

The moisture content of the flakes was measured from 0.3 g of sample employing an EM 120-HR moisture analyser (Precisa Gravimetrics AG, Dietikon, Switzerland). Measurements were performed in triplicate, results are expressed in percent wet basis.

#### Water activity

The water activity of the flakes was measured using an AquaLab Series 4 aw meter (Decagon Devices, Inc., Pullman, WA) after the samples were stabilized at 25 °C for 30 min^31^. The measurements were performed in triplicate.

#### Contact angle (CA)

For contact angle (CA) measurements of the flakes a MobileDrop contact angle meter (Kruss, Hamburg, Germany) was used, three measurements were performed with a measuring drop of 4.5 µL, and the contact angle was measured at 1 s after the release of the droplet.

#### Color

The color of the fresh nanoemulsions and flakes was measured. The measurements were carried out with a ColorQuest XE (HunterLab, U.S.A). The results were expressed in accordance with the CIELAB system with reference to illuminant C and a visual angle of 10°^32^. The colorimeter was calibrated with a black and white standard patterns. Hue (h_ab_) was determined using Eq. (1).

1 $${\text{h}}_{{{\text{ab}}}} = {\text{ arctan }}\left( {{\text{b}}^{*}/{\text{a}}^{*}} \right)$$

In this case, b* is the yellow/blue coordinate, and a* the red/green coordinate. Total color difference (ΔE*) regarding fresh nanoemulsions was calculated as follows^33^.

2 $$\Delta {\text{E}}^{*} = [(\Delta {\text{L}}^{*})^{{2}} + [(\Delta {\text{a}}^{*})^{{2}} + [(\Delta {\text{b}}^{*})^{{2}} ]^{{{1}/{2}}}$$

The ΔL*, Δa* and Δb* variables are the difference values between a reference color and the color of the sample; ΔL* represents the difference of lightness, Δa* represents the difference of red/green coordinate, and Δb* represents the difference of yellow/blue coordinate. The values used to determine whether the total color difference was appreciable by the human eye were the following^34^:

- ΔE* < 1 color differences are not obvious for the human eye.
- 1 < ΔE* < 3 color differences are not appreciated by the human eye.
- ΔE* > 3 color differences are obvious for the human eye.

Measurements were performed in triplicate in three different flakes obtained by RW drying.

#### Scanning electron microscopy (SEM)

The analysis of microstructure of flakes was performed using a scanning electron microscope (Phenom Pro, Cecoltec Ltda, Bogotá, Colombia) at an acceleration voltage of 5 kV. The samples of 2 mm wide were placed on the scanning electron microscope slides with the aid of colloidal silver and a magnification of 4000x was used for the analysis.

#### Image analysis

Images of the flakes of 2048 × 2176 pixels were captured using an electronic microscopy and stored as bitmaps in a gray scale with brightness values between 0 and 255 for each pixel constituting the image. A generalization of the box counting method was used to evaluate the fractal dimension of the images (FDt). In this work, the shifting differential box-counting method (SDBC)^35^ was used to evaluate the fractal dimension of texture of SEM images using the ImageJ 1.34 software. Four different images at the same magnification (4000×) were evaluated for each flake HOPO nanoemulsion. The size of the crops was 67.81 μm × 67.81 μm. The texture parameters, contrast, correlation, inverse difference moment (IDM), and entropy of SEM images were evaluated using the GLCM and surface plot tools of ImageJ.

The textural feature *contrast* is a measure of the intensity contrast between a pixel and its neighbour over the whole image. It measures the local variation in the GLCM. Contrast is 0 for a constant image^34^. The textural feature *correlation* is a measure of how correlated a pixel is to its neighbour over the whole image. Its range lies between − 1 and + 1. Also, the correlation is 1 or − 1 for a perfectly positively or negatively correlated image. Correlation measures the joint probability of occurrence of pixel pairs of GLCM^36^. The textural feature *inverse difference moment* (IDM) measures the texture uniformity or orderliness of an image but normalized for distance. As defined by Yang and collaborators, higher inverse difference moment values indicate a variation in image contrast^37^, that is, greater heterogeneity of the flakes texture. The textural feature *entropy* is a statistical measure of randomness that can be used to characterize the texture of the input image^38^.

### Statistical analysis

The statistical analysis was performed using the Box-Behnken optimization experimental design methodology in the Design Expert Software Version 10.1.0 (Stat-Ease Inc., MN, USA). Quadratic models were used to express the response variables as a function of the independent factors, where A, B, C, and D, are the coded values of the drying temperature, the sample thickness, pressure, and formulation, respectively. A statistical significance test was used in the total error criteria with a confidence level of 95%. The significant terms in the model were found through analysis of variance (ANOVA). The fit of the model was evaluated by the R^2^ value.

The graphic and numerical optimization of the Design Expert software was used for response optimization. Two optimisation systems (Opt A and Opt B) were formulated; one for a nanoemulsion made from formulation A (Opt A) and the other for a nanoemulsion made with formulation B (Opt B), both having minimum moisture and water activity (a_w_) after RW drying.

## Results and discussion

### Moisture

A sample’s moisture during drying is the ratio of a sample’s mass of water after drying to a sample’s total mass. Table 2 gives analysis of variance (ANOVA) results for moisture response. The results were fitted to a quadratic model, giving 0.81 R^2^. Table 2 shows that the model was significant (*p* < 0.05) and had 0.0584 lack of fit (*p* > 0.005); such result indicated that the model was suitable and could thus predict the moisture content for flakes from RW dried-nanoemulsions, prepared from a determined concentration of buttermilk, starch or gum Arabic and oil. The flakes’ moisture content ranged from 1.8 to 5.65% (Table 1).

**Table 2:** ANOVA for the adjusted variables to the Box-Behnken design: moisture, a_w_, CA, h_ab_, ΔE*, FDt, contrast, correlation, IDM and entropy for flakes of HOPO nanoemulsions.

	Moisture (%)			a_w_			CA (°)			h_ab_ (°)			∆E*		
	SS	df	p-value	SS	df	*p-*value	SS	df	*p-*value	SS	df	p-value	SS	df	p-value
Model	21.691	19	0.014	0.0602	19	0.0283	44.506	19	0.0287	582.031	19	0.0047	361.620	13	0.8568
A	12.616	1	< 0.001	0.0264	1	0.0003	16.091	1	0.0007	0.1585	1	0.6504	0.2055	1	0.8408
B	1.096	1	0.099	0.0001	1	0.7707	0.0057	1	0.7991	112.341	1	0.0016	0.6546	1	0.7203
C	0.500	1	0.253	0.0001	1	0.7721	0.0034	1	0.8431	155.746	1	0.0004	94.393	1	0.1831
D	1.190	1	0.087	0.0005	1	0.5353	0.0049	1	0.8141	41.644	1	0.0325	12.809	1	0.6170
AB	0.010	1	0.872	9.59. × 10^–7^	1	0.9773	0.2546	1	0.1049	0.0181	1	0.8778	0.7426	1	0.7030
AC	0.907	1	0.130	0.0011	1	0.3391	0.0892	1	0.3221	0.6954	1	0.3486	19.124	1	0.5418
AD	0.064	1	0.676	0.0001	1	0.7448	0.2214	1	0.1282	0.3666	1	0.4929	0.6601	1	0.7192
BC	0.578	1	0.221	0.0002	1	0.6778	0.1505	1	0.2038	0.2613	1	0.5617	0.7783	1	0.6963
BD	0.223	1	0.439	0.0005	1	0.5182	0.0122	1	0.7100	0.3028	1	0.5325	141.568	1	0.1068
CD	1.197	1	0.086	0.0001	1	0.7738	0.0001	1	0.9696	23.871	1	0.0940	16.635	1	0.5691
A^2^	1.172	1	0.089	0.0116	1	0.0066	15.175	1	0.0008	0.1066	1	0.7099	0.8942	1	0.6758
B^2^	0.009	1	0.874	0.0005	1	0.5044	0.0170	1	0.6610	0.2300	1	0.5859	27.530	1	0.4651
C^2^	0.086	1	0.628	0.0009	1	0.3915	0.1142	1	0.2649	117.771	1	0.0013	0.5959	1	0.7326
ABD	0.896	1	0.133	0.0083	1	0.0175	0.0226	1	0.6137	0.3240	1	0.5187	–		–
ACD	0.346	1	0.338	0.0020	1	0.2031	0.1528	1	0.2006	70.846	1	0.0079	–		–
BCD	0.147	1	0.529	0.0004	1	0.5822	0.0566	1	0.4274	0.3270	1	0.5169	–		–
A^2^B	–	–	–	–	–	–	–	–	–	34.180	1	0.0495	–		–
A^2^D	0.003	1	0.931	0.0010	1	0.3568	0.0479	1	0.4645	34.180	1	0.0495	–		–
B^2^D	0.953	1	0.122	0.0016	1	0.2587	0.0021	1	0.8784	0.3133	1	0.5256	–		–
C^2^D	0.570	1	0.223	0.0045	1	0.0672	0.1028	1	0.2892	0.9610	1	0.2734	–		–
Lack of fit	3.522	6	0.058	0.0085	6	0.2828	0.3734	6	0.7162	20.427	6		495.597	12	0.7475
Pure error	1.396	8		0.0075	8		0.8123	8		83.086	8		496.984	8	
R^2^	0.815			0.7902			0.7896			0.8490			0.2670		

	FDt			Contrast			Correlation			IDM			Entropy		
	SS	df	*p-*value	SS	df	*p-*value	SS	Df	*p-*value	SS	df	*p-*value	SS	df	*p-*value
Model	0.0311	13	< 0.0001	760,691.83	13	< 0.001	5.77x^10–7^	13	0.0002	0.0006	13	0.0004	1.0449	13	3.71 × 10^–4^
A	0.0126	1	< 0.0001	440,783.29	1	< 0.001	3.16x^10–7^	1	< 0.001	0.0003	1	< 0.001	0.4446	1	< 0.001
B	0.0004	1	0.2241	3557.04	1	0.4760	1.14 × 10^–9^	1	0.6937	2.0 × 10^–5^	1	0.1413	0.0011	1	0.7870
C	0.0008	1	0.1029	244.67	1	0.8508	7.20 × 10^–9^	1	0.3278	1.3 × 10^–6^	1	0.7058	0.0947	1	0.0193
D	2.28 × 10^–5^	1	0.7786	416.98	1	0.8061	4.08 × 10^–11^	1	0.9406	1.5 × 10^–6^	1	0.6766	0.0001	1	0.9236
AB	4.03 × 10^–6^	1	0.9059	126.59	1	0.8924	1.13 × 10^–9^	1	0.6952	7.0 × 10^–6^	1	0.3775	7. × 10^–6^	1	0.9827
AC	0.0003	1	0.3045	28,624.14	1	0.0526	4.17 × 10^–9^	1	0.4543	3.2 × 10^–5^	1	0.0685	0.0154	1	0.3172
AD	1.52 × 10^–5^	1	0.8184	318.22	1	0.8302	2.19 × 10^–10^	1	0.8629	1.1 × 10^–5^	1	0.2817	0.0068	1	0.5037
BC	4.78 × 10^–5^	1	0.6845	933.75	1	0.7137	4.26 × 10^–10^	1	0.8097	2.5 × 10^–6^	1	0.5941	0.0016	1	0.7457
BD	0.0006	1	0.1543	301.61	1	0.8346	1.02 × 10^–8^	1	0.2474	1.4 × 10^–5^	1	0.2164	0.0304	1	0.1652
CD	0.0002	1	0.4586	500.51	1	0.7881	1.20 × 10^–9^	1	0.6867	6.2 × 10^–8^	1	0.9330	0.0301	1	0.1669
A^2^	0.0138	1	< 0.0001	273,923.46	1	0.0000	2.35 × 10^–7^	1	< 0.0001	0.0002	1	< 0.0001	0.3929	1	< 0.0001
B^2^	2.1 × 10^–5^	1	0.7890	1598.71	1	0.6316	6.46 × 10^–13^	1	0.9925	1.3 × 10^–6^	1	0.6969	0.0173	1	0.2892
C^2^	0.0028	1	0.0048	12,319.32	1	0.1915	9.15 × 10^–10^	1	0.7244	9.6 × 10^–7^	1	0.7423	0.0016	1	0.7417
ABD	–	–	–	–	–	–	–	–	–	–	–	–	–	–	–
ACD	–	–	–	–	–	–	–	–	–	–	–	–	–	–	–
BCD	–	–	–	–	–	–	–	–	–	–	–	–	–	–	–
A^2^B	–	–	–	–	–	–	–	–	–	–	–	–	–	–	–
A^2^D	–	–	–	–	–	–	–	–	–	–	–	–	–	–	–
B^2^D	–	–	–	–	–	–	–	–	–	–	–	–	–	–	–
C^2^D	–	–	–	–	–	–	–	–	–	–	–	–	–	–	–
Lack of fit	0.0020	12	0.9433	43,465.57	12	0.9639	2.63 × 10^–8^	12	0.9981	0.0001	12	0.9769	0.1401	12	0.7864
Pure error	0.0036	8		91,366.54	8		1.17 × 10^–7^	8		0.0001	8		0.1526	8	
R^2^	0.8468			0.849			0.8011			0.7776			0.7812		

Drying temperature was the variable which significantly affected (*p* < 0.05) the resulting moisture pattern; the higher the drying temperature, the lower the moisture content in the resulting flake. Even though the pattern was the same for both formulations, formulation B had lower moisture content at the same temperature than that for formulation A. The above could be due to the difference in wall material in both formulation (formulation A was prepared with corn starch and formulation B with gum Arabic), the molecular weight of wall materials impact its capacity of absorb water, affecting directly the moisture content of the sample. In the supplementary file Table S1 are shown the equations describing the pattern of moisture content for both formulations.

The samples’ moisture content was only affected by water temperature during drying; this was the variable directly influencing RW drying’s three most important aspects: heat emission by the drying source (hot water), propagation of heat by the medium (Mylar polyester film) and the heat absorbed by the product (emulsion). Zotarelli et al.^39^ have reported how using Mylar film led to more than 40% transmittance regarding maximum heat emission (60 °C to 100 °C) by water radiation (10–100 W/m^2^); such values were more than sufficient for good heat transfer by radiation between the heat source and the water in the emulsion^40^. This data suggested the effectiveness of heat transfer during RW drying.

The forgoing highlighted the fact that moisture content varied from 1.8 to 5.65% for the proposed temperature interval and gave favourable values for RW dried products, as values below 15% prevent antimicrobial growth, increase a sample’s structural stability and retard deterioration reactions (i.e. sugar crystallisation and non-enzymatic browning)^41^.

### Water activity

Water activity (a_w_) is an important parameter which can influence food shelf-life since it reflects the amount of water available for chemical reactions and microorganism growth (i.e. bacteria, fungi and yeasts)^42^. Table 2 gives ANOVA results for water activity (a_w_) response. The results were fitted to a quadratic model (0.79 R^2^). Table 2 shows that the model was significant (*p* < 0.05) and had a 0.28 lack of fit, indicating that the proposed model’s a_w_ provided a suitable fit and could thus fit and predict an evaluable water activity pattern for flakes from RW-dried nanoemulsions.

Only temperature had a significant impact on water activity pattern (*p* < 0.05); at higher temperatures, less water activity. The temperature squared (A^2^) value also significantly affected a_w_ (*p* < 0.05); Table S1 of Supplementary file shows the equations describing water activity pattern for each formulation.

This study emphasised the relationship between drying temperature and a_w_, as increasing the drying temperature led to a reduction in the flakes’ a_w_; this could have been related to the fact that more water would have been evaporated at higher temperatures^43^. However, no mention was found in the pertinent literature of variation concerning drying temperature and its effect on a_w_, as most studies compare RW with other drying methods at a single temperature or vary drying times or sample thickness.

Drying is one of the most efficient methods for conserving food, as reducing a_w_ reduces microbial growth and decreases the speed of degradation reactions, the latter being of great interest for drying products having antioxidant activity. Around 0.3 a_w_ values were obtained for the flakes (Table 1) which authors like Pavan et al.^24^ (whose group has dried açai pulp) have considered a standard value for pulp dried by any method, since water having higher a_w_ is sufficient to behave like a solvent, thereby increasing the mobility of products used in/available for degradation reactions^13^. Other authors have focused more on the relationship of a_w_ with moisture and storage temperature which are very important aspects when considering food conservation^12,13,39^.

### Contact angle

Contact angle is a measurement quantifying a substance’s hydrophilic or hydrophobic behaviour as an important parameter when analysing surfaces’ interaction with water; a smaller angle measurement indicates hydrophilic behaviour whilst a greater angle indicates a hydrophobic pattern^44^.

ANOVA was used for ascertaining quadratic fit (Table 2) and square root transformation (Supplementary file Table S1) of data regarding contact angle (0.78 R^2^). Table 2 shows that the model was significant (*p* < 0.05) and had 0.72 lack of fit, suggesting the model’s great reliability or describing its pattern and possibly acting as a tool for analysing the contact angle of RW dried nanoemulsion flakes in similar conditions.

Like a_w_, temperature (A) and its quadratic relation (A^2^) were the variables which significantly affected the contact angle. Contact angle varied from 15.72° to 31.99° (Table 1); a sample’s lower drying temperature meant a smaller contact angle whilst formulation A had a slightly more hydrophobic pattern (CA = 24°) than formulation B (B = 22°), given the same temperature and thickness values. This pattern could have been due to the chemical nature of gum Arabic (Formulation B) since its structure (consisting of a branched carbohydrate chain (d-glucuronic acid, l-rhamnose, d-galactose and l-arabinose) has glycoproteins bound by covalent linkages, making it a highly hydrophilic molecule^45^. Amylose, which makes up 50% of corn starch, consists of glycosidic bonds producing a simple helix whose interior only consists of hydrogen atoms, thereby giving a lipophilic pattern^46^. In the supplementary file Table S1 are shown the equations describing contact angle pattern for both formulations.

Studying contact angles is closely linked to the science of materials and polymers, so studying it in the agri-food industry has been very limited and (to the best of our knowledge) no research has yet been published on contact angles on surfaces obtained from RW-dried matrices.

Authors who have researched food area-related contact angles have based their work on studying the properties of materials’ surfaces in response to contact with liquid foodstuffs (i.e. interfacial interactions). Their usefulness as a mechanism for predicting interactions between liquids and solid surfaces (i.e. biofilms, membranes, heat interchange or packed food surfaces) has been studied by authors like Güleç et al.^47^. Such authors have studied the hydrophilicity, hydrophobicity and surface free energy of three different materials used in the food packaging industry (glass, polyethylene and stainless steel); they studied contact angle variation regarding surface and interfacial tension to ascertain the effects of packaging surface contaminants on food and microorganism growth. Other studies have focused more on predicting food packaging surface structure by using mathematical relationships to modify surfaces to control contact angles and surface energy^48,49^.

### Color

Table 2 gives ANOVA results for variables h_ab_ and ΔE^*^. The table shows that even though the lack of fit was p > 0.05 in all cases, the model did not fit (*p* > 0.05) variable ΔE^*^ (Table 2, in red), indicating that it might not accurately predict such flake color parameters. The results for color parameter h_ab_ fit a quadratic model R^2^:0.85 for h_ab_.

Variable h_ab_ was significantly affected (*p* < 0.05, Table 2) by flake thickness, microfluidization pressure and formulation. Increasing flake thickness significantly reduced (*p* < 0.05) h_ab_, this could be due to the exposure of the sample film to the drying temperature; lower thickness values, less layers to receive the heat transfer, which results in a yellowing of the sample. On the other hand, increasing microfluidization pressure significantly increased (*p* < 0.05) h_ab_ wich could be due to the organization of the droplets of the sample, that at higher microfluidization pressure, lower the droplet size of the emulsion^50–54^. The same happened for formulation B, h_ab_ values were higher than those for formulation A; in this case, the main difference between formulation A and B was the wall material (corn starch and gum Arabic, respectively), letting conclude that gum Arabic preserve better the color of the original food. The flakes h_ab_ values ranged from 73.24° to 78.80° (Table 1), related to high purity regarding yellow tonality since such values were close to 90°^55^.

Table 3 gives the fresh nanoemulsions’ color parameters (L*, a*, b*, h_ab_). Color difference (ΔE*) values regarding flakes and nanoemulsions before RW drying varied from 3.81 to 13.29 (Table 1), i.e. an obvious color difference between flakes and fresh nanoemulsion for the human eye (ΔE^*^ > 3)^34^. Such results showed that the temperature of RW drying of a nanoemulsion does produce important changes in parameters (L*, a*, b*), also the pressure of microfluidization which led to encapsulation of the oil, thereby producing high color differences obtained at lower pressure values and higher temperatures. Such difference may have been due to carotene degradation in palm oil (i.e. natural antioxidants controlling color^56^ during drying as it has been widely reported that they are temperature-sensitive (i.e. the higher the temperature, the greater carotene degradation, especially when 60 °C has been exceeded)^57–59^.

**Table 3:** Lightness (L*), color coordinate a*, color coordinate b*, and hue (h_ab_), for fresh nanoemulsions of HOPO.

Pressure (psi)	L*		a*		b*		h_ab_ (°)	
	A	B	A	B	A	B	A	B
0	63.4 (0.4)	64.1 (0.6)	15.1 (< 0.01)	14.6 (0.2)	80.3 (1.4)	81.0 (2.3)	79.3 (0.2)	79.8 (0.2)
10,000	68.5 (0.6)	68.7 (0.1)	11.3 (0.2)	11.8 (0.2)	73.8 (1.9)	74.1 (1.5)	81.3 (0.4)	80.9 (< 0.01)
20,000	66.3 (1.3)	66.7 (1.0)	13.5 (2.1)	12.4 (0.4)	80.9 (3.6)	75.6 (3.0)	80.5 (1.0)	80.7 (0.6)

The values in parenthesis are the standard deviations.

### Scanning electron microscopy (SEM)

**Figure 2:**
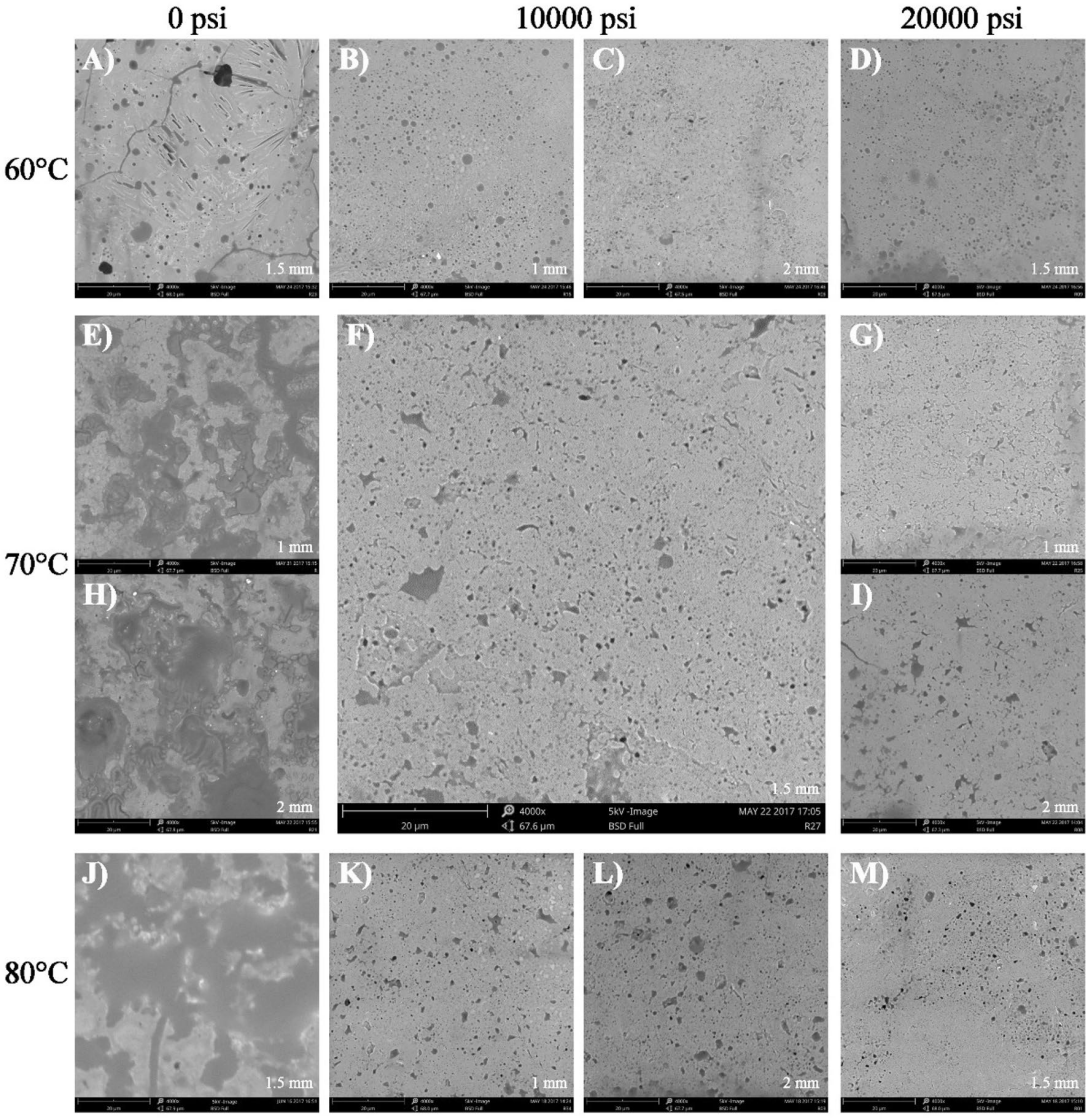
Scanning electron microscopy micrographs for flakes of HOPO nanoemulsions elaborated using formulation A. Magnification: 4000x. The labelling of the imagen (letters A to M) was the order in which SEM images were took. Processed with ImageJ 1.34 software (https://imagej.nih.gov/ij/notes.html).

**Figure 3:**
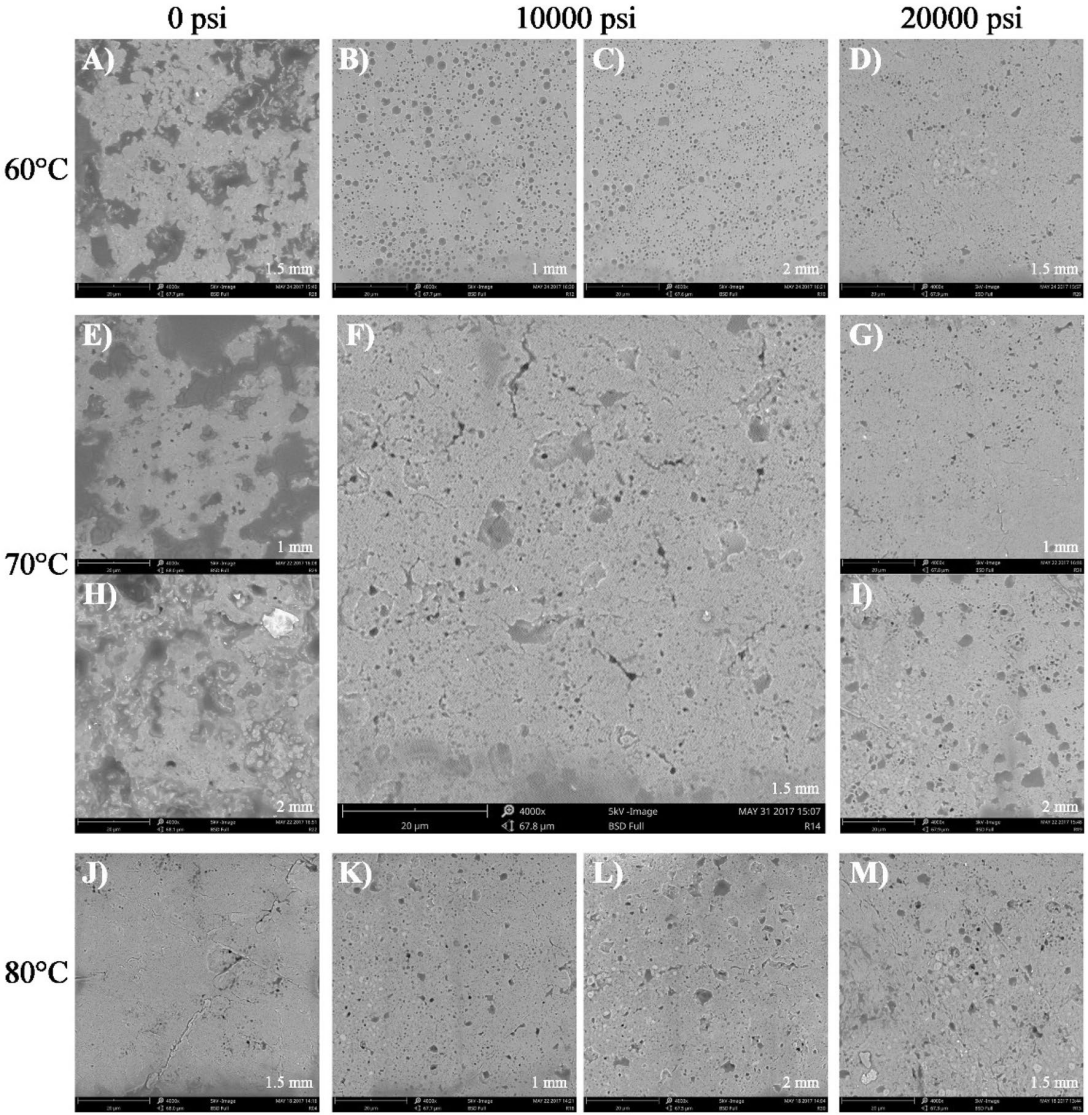
Scanning electron microscopy micrographs for flakes of HOPO nanoemulsions elaborated using formulation B. Magnification: 4000x. The labelling of the imagen (letters A to M) was the order in which SEM images were took. Processed with ImageJ 1.34 software (https://imagej.nih.gov/ij/notes.html).

Figures 2 and 3 show SEM microstructures of flakes from formulations A and B for every drying temperature and microfluidization pressure used in the study; the porous structure of flakes obtained by drying HOPO nanoemulsions from both formulations was revealed by microstructural study. Nanoemulsions which were not microfluidized (0 psi) had a less homogeneous structure, having larger pores which joined together forming large holes; pore size became reduced when increasing microfluidization pressure. It would thus seem that using higher microfluidization pressure gives more homogeneous microstructure nanoemulsions, as RW drying results in flakes having a more uniform structure, with smaller sized pores, since the continuous phase is more homogeneous in the emulsion. Increased nanoemulsion homogeneity related to decreased particle size when increasing microfluidization pressure has already been reported^19,20^.

### Image analysis

Table 2 gives ANOVA results for FDt, contrast, correlation, IDM and entropy response. The results for all texture parameters analysed here fit a quadratic model; texture parameters R^2^ were 0.85 for FDt and contrast, 0.8 for correlation and 0.78 for IDM and entropy. Table 2 shows that the model was significant (*p* < 0.05) and had > 0.05 lack of fit, thereby indicating its suitability for predicting the texture parameters which would be obtained when a nanoemulsion prepared at a specific microfluidisation pressure was dried at a determined temperature and lamina thickness.

Texture FDt analysis regarding drying temperature and drying temperature squared (A^2^) was statistically significant (*p* < 0.05) (Table 2). Increased temperature produced a significant reduction (*p* < 0.05) in the flakes’ FDt for both formulations. Lower FDt values were related to more homogenous and regular surfaces. FDt was also affected by the microfluidization pressure squared (C^2^) used in preparing the nanoemulsion.

The values obtained came within the range reported to date for food surface images^60–63^. The pertinent literature states that low FDt values at high drying temperatures could be associated with images having a smooth fractal texture, while high FDt values could be related to images having rough fractal texture^62^. Hernández-Carrión et al.^63^ found that Lamuyo red pepper FDt was lower when less structural damage was caused by high hydrostatic pressure. Such results would explain why lower FDt values were obtained at higher microfluidization pressures, related to a more homogeneous and regular structure, as can be seen in the microstructure study. Authors like Aragón-Rojas et al.^64^ have established a relationship between freeze-dried powders’ FDt and moisture. Such authors have stated that freeze-dried powders have greater surface area exposed to the environment when their surface is more rugged (higher FDt values) and their moisture will thus be higher. This would explain the results obtained when analysing the flakes’ moisture, as flakes with higher values of FDt also had the higher values of moisture content.

Texture contrast, correlation and IDM parameters were significantly affected (*p* < 0.05) by RW drying temperature, as well as such variable squared (A^2^) (Table 2). Increased drying temperature led to a significant reduction (*p* < 0.05) in image contrast, whilst producing a significant increase (*p* < 0.05) in correlation and IDM for both formulations. Such results indicated that increasing nanoemulsion drying temperature led to a more homogenous and regular texture of flakes after drying.

A high contrast value indicates a high degree of local variation^65,66^, this being typical of rougher and more heterogeneous surfaces. This would explain the higher contrast values for rougher surfaces observed at low drying temperatures and the lower values for a more homogenous surface at high drying temperatures. It is known that IDM values indicate the degree of image contrast variation and high IDM values can be associated with homogeneous images^65^, such as images obtained at high drying temperatures. An increase in temperature tended to increase the IDM which was related to the more homogenous structures observed at high drying temperatures.

Similar results were obtained by Barrera et al.^67^ when evaluating mechanical damage to wheat starch granules. They concluded that damaged granule surface had lower IDM values than those for native starch granules, suggesting that the mechanical process decreased IDM. Hernández-Carrión et al.^63^ evaluated structural damage to Lamuyo red pepper caused by high hydrostatic pressure treatment and pasteurisation; they found that treatment causing more structural damage to red pepper tissue had lower IDM values and higher contrast values than those causing lower structural damage, thereby suggesting that structural damage decrease IDM values and increase contrast values. The above suggest that drying nanoemulsions at low temperatures could a more heterogeneous flake structure as the IDM values obtained for these temperatures decreased.

Entropy was significantly affected (*p* < 0.05) by drying temperature and the microfluidization pressure of nanoemulsions (Table 2). Increased drying temperature and microfluidization pressure led to a significant reduction (*p* < 0.05) of the flakes’ entropy for both formulations. The higher entropy values obtained at low drying temperature and microfluidization pressure could have been related to their structure’s greater heterogeneity^37^ since more complex images are associated with higher entropy values^65,67^.

The lower entropy values found at high drying temperatures and microfluidization pressures could have been related to their structures’ greater homogeneity^37,65^. Hernández-Carrión et al.^63^ when evaluating structural damage to Lamuyo red pepper subjected to high hydrostatic pressure treatment and pasteurization stated that treatments causing more structural damage to red pepper tissue had higher entropy values than those causing lower structural damage, suggesting that structural damage increased entropy.

Increased nanoemulsion drying temperature was thus mainly responsible for improving the parameters regarding image texture as a more homogeneous and regular texture was thereby obtained at higher temperatures. Table S1 of Supplementary file, gives the prediction equations for the texture parameters from the model’s parameters for formulations A and B.

### Optimisation

**Figure 4:**
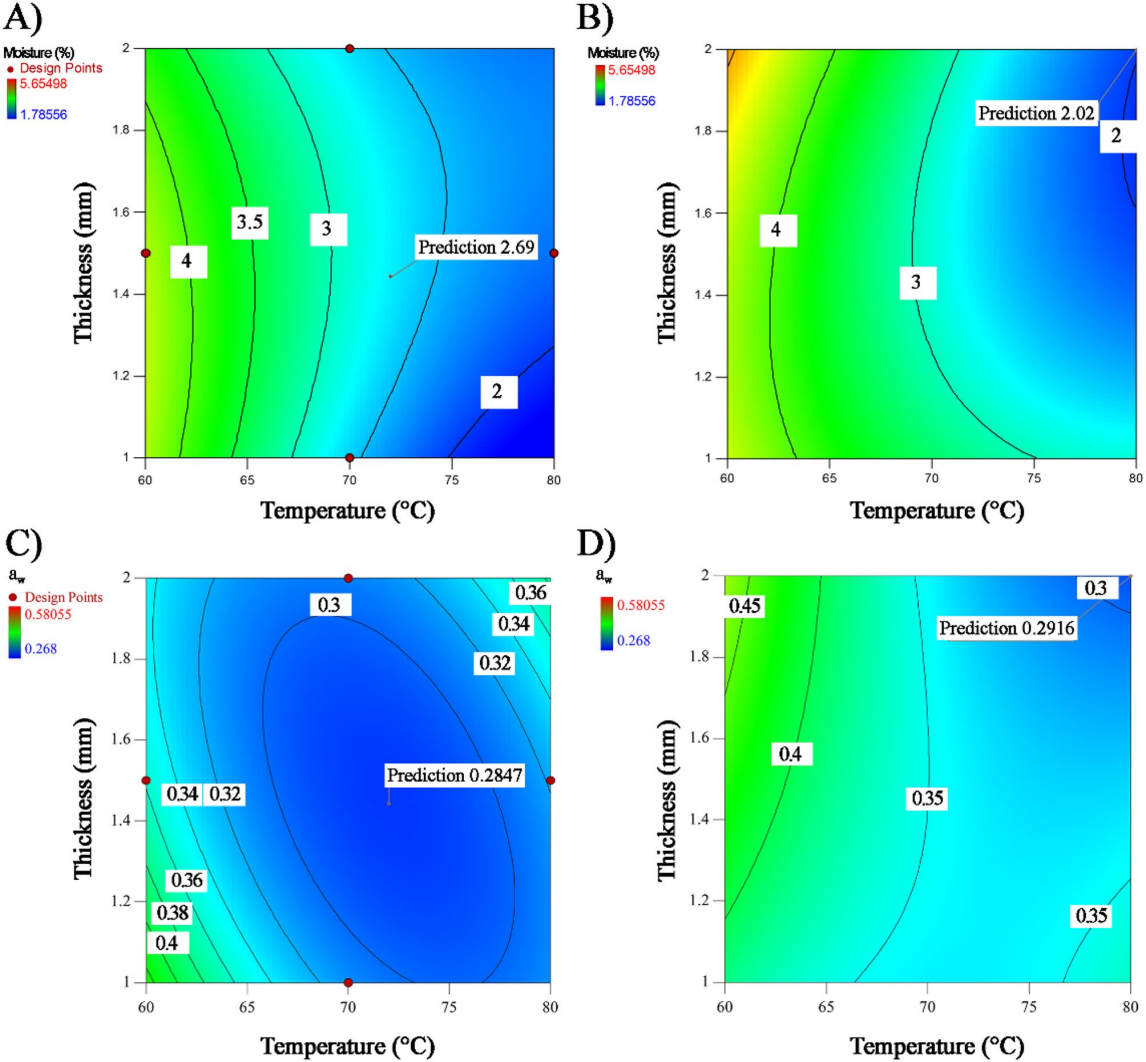
Isoplots for adjusted variables the Box-Behnken optimal design: (**A**) Moisture content for Formulation A, (**B**) Moisture content for Formulation B, (**C**) water activity for Formulation A, and (**D**) water activity for formulation B. Obtained from: Design Expert Software Version 10.1.0 (Stat-Ease Inc., MN, USA, https://www.statease.com/software/design-expert/ ).

Table 4 and Fig. 4 show that high drying temperature (72 °C), intermediate thickness (1.44 mm) and high microfluidization pressure (20,000 psi) must be used for obtaining flakes from nanoemulsions made from formulation A having low moisture content of 2.69% and a_w_ of 0.2847. Higher drying temperature (80 °C), greater thicknesses (2 mm) and lower microfluidization pressure (16,237 psi) would have to be used with formulation B for ensuring low moisture (2.02%) and a_w_ (0.2916).

**Table 4:** Experimental optimum conditions obtained by the Box-Behnken optimization design for moisture and a_w_: experimental values vs values of prediction equations.

	Temperature (°C)	Thickness (mm)	Pressure (psi)	Formulation	Moisture (%)		a_w_	
					Experimental	Model	Experimental	Model
OptA	72.00	1.44	20,000	A	2.85	2.69	0.2937	0.2847
OptB	80.00	2.00	16,237	B	2.08	2.02	0.2766	0.2916

High drying temperatures and microfluidization pressures were thus mainly responsible for obtaining flakes having low moisture and a_w_; flakes having lower moisture and aw were obtained from nanoemulsions made from formulation B than formulation A.

It should be stressed that optimal solution desirability was > 92% and moisture and a_w_ experimental values were very close to those estimated by the model’s prediction equations (Table 4). Maximum error was 5.76% for optimal nanoemulsion A moisture whilst a 2.70% minimum error was recorded for optimal nanoemulsion B moisture.

## Conclusions

Using response surface methodology for studying the effect of emulsion formulation drying conditions resulted in mathematical models for predicting the pattern of the variables analysed in this work. These results suggested that RW drying, and microfluidization as a nanoencapsulation technique of high-oleic palm oil, could lead to producing dried products in the form of flakes, however, it causes an effect in the color of the emulsion which could be perceived by the human eye. In this case, they were formulated with biopolymers such as corn starch and gum arabic, having high oil content, allowing optimum physical properties represented in lower moisture content and lower water activity, mostly affected by the pressure of microfluidization and drying temperature. For the best of our knowledge, this is the first article that analyses the microstructure of flakes containing oil using RW, that was affected only by the drying temperature. These results provide evidence of new ways of dried structures with high oil contents different from powders. This paper shows the importance of develop technologies and processes that enhance products and allow the addition of compounds at cheaper prices than those that can be offered by technologies such as freeze-drying.

## Supplementary Information

Supplementary Information.
