# Supplementary material for: The effect of process variables on the physical properties and microstructure of HOPO nanoemulsion flakes obtained by refractance window
Source: Sci Rep. 2021 Apr 30;11:9359. doi: 10.1038/s41598-021-88381-7 (PMC8087804; doi:10.1038/s41598-021-88381-7)
Supplement: Supplementary file 1 — Supplementary Information. [file 41598_2021_88381_MOESM1_ESM.docx]

**Supplementary File of :** **The effect of process variables on the physical properties and microstructure of HOPO nanoemulsion flakes obtained by refractance window**

M Hernández-Carrión^a^, M Moyano-Molano^a^, L Ricaurte^a^, A Clavijo-Romero^a^ and MX Quintanilla-Carvajal^a*^

**Table S1**. Equations for Moisture, a_w_, CA, L^*^, a^*^, h_ab_, FDt, contrast, correlation, IDM, and entropy for flakes of HOPO nanoemulsions.

| **Equations for** | **Formulation A** | | **Formulation B** | |
| --- | --- | --- | --- | --- |
| **Moisture =** | 33.933 -0.720x[**A**] -2.131x[**B**] - 8.987x10^-6^ x [**C**] + 0.060x[**AB**] -1.289x10^-6^x [**AC**] + 8.083x10^-5^x [**BC**] + 3.909 x 10^-3^x[**A^2^**] - 1.213x[**B^2^**] - 1.589x10^-9^x [**C^2^**] | | | 18.136-0.414 x [**A**] +0.182 x [**B**]+ 3.141x10^-4^ x [**C**] -0.074x[**AB**] -5.445x10^-6^ x [**AC**] +2.666x10^-5^x[**A**D] +3.550x10^-3^x[**A^2^**] +1.478x [**B^2^**] +3.614 x 10^-9^x[**C^2^**] |
| **√a_w_ =** | 4.281 - 0.084x[**A**] -0.715x[**B**] - 1.376x10^-4^ x [**C**] + 6.365x10^-3^ x [**AB**] +2.778x10^-7^ x [**AC**] + 3.319x10^-7^x[**BC**] + 4.817x10^-4^x[**A^2^**] + 0.087x[**B^2^**] - 3.341x10^-10^x[**C^2^**] | | 1.313 - 0.030x[**A**] -0.564x[**B**] -3.866x10^-6^x[**C**] - 6.503x10^-3^x[**AB**] -4.128x10^-8^x[**AC**] -2.360x10^-6^ x [**BC**] + 2.598x10^-4^ x[**A^2^**] - 0.029 x [**B^2^**] + 1.281x10^-10^x[**C^2^**] | |
| **√CA (°) =** | 30.558 - 0.725x[**A**] - 2.638x[**B**] + 1.577x10^- 4^x [**C**] + 0.046x[**AB**] - 2.438 x 10^- 6^ x [**AC**] + 1.061 x 10^- 4^x[**BC**] + 4.999 x 10^- 3^x[**A^2^**] - 0.242x[**B^2^**] - 4.979x10^– 11^x[**C^2^**] | | 21.676 - 0.486x[**A**] -1.752x[**B**] -4.261x10^-4^x[**C**] + 0.025 x [**AB**] +3.258x10^-7^x[**AC**] + 4.425x10^-4^x[**BC**] +3.491x10^-3^x[**A^2^**] - 0.117x[**B^2^**] -2.269x10^-9^x[**C^2^**] | |
| **h_ab_* =** | 35.129 + 1.194x[**A**] -3.530x[**B**] +7.789 x10^-4^x[**C**] -0.050x[**AB**] 6.462x10^-6^x[**AC**] + 7.658x10^-4^x[**BC**] -7.496x10^-3^x[**A^2^**] +1.432x[**B^2^**] - 1.520x10^-8^x[**C^2^**] | 115.958-0.929 x [**A**]-3.177 x [**B**]-6.297x10^-4^ x [**C**]+0.031 x [**AB**]+1.236x10^-4^x [**AC**]-4.286x10^-6^x [**BC**] +5.246x10^-3^x[**A^2^**] -0.110x[**B^2^**] - 8.445x10^-9^x[**C^2^**] | | |
| **FDt =** | 4.716 - 0.059x[**A**] - 0.027x[**B**] +9.802x10^-6^x[**C**] - 1.419x10^-4^x[**AB**] - 6.247x10^-8^x [**AC**] - 4.888x10^-7^x[**BC**] + 4.044x10^-4^x[**A^2^**] + 6.267x10^-3^x[**B^2^**] - 1.831x10^-10^x [**C^2^**] | 4.673 -0.059x [**A**] - 2.09x10^-3^ x [**B**] +9.168x10^-6^x [**C**]-1.419x10^-4^x [**AB**]-6.248x10^-8^ x [**AC**]-4.888x10^-7^x [**BC**] + 4.044x10^-4^x [**A^2^**]+6.267x10^-3^x [**B^2^**]- 1.831x10^-10^x [**C^2^**] | | |
| **Contrast =** | -10234.239 -261.476x[**A**]- 169.763x[**B**] +0.047x[**C**]- 0.796x[**AB**]-5.982x10^-4^x[**AC**] + 2.161x10^-3^x[**BC**] +1.804x[**A^2^**] + 55.114x[**B^2^**] -3.825x10^-7^x [**C^2^**] | 10274.806- 262.368 x [**A**]-152.396 x [**B**]+0.046 x [**C**] -0.796 x [**AB**]- 5.982x10^-4^x [**AC**] +2.161x10^-3^ x [**BC**] +1.803x [**A^2^**] + 55.114 x [**B^2^**]- 3.825x10^-7^ x [**C^2^**] | | |
| **Correlation =** | 0.008 +2.423x10^-4^x[**A**]- 8.125x10^-4^x[**B**]-1.462x10^-8^x[**C**]+2.378x10^-6^x[**AB**]+ 2.282x10^-10^x[**AC**]-1.459x10^-9^x[**BC**]-1.669x10^-6^x[**A^2^**]-1.107x10^-6^x[**B^2^**] +1.043x10^-13^ x[**C^2^**] | -0.008 + 2.41x10^-4^ x [**A**]-1.820x10^-4^ x [**B**]-1.289x10^-8^ x [**C**]+2.378x10^-6^x [**AB**] + 2.282x10^-10^ x [**AC**] -1.459x10^-9^ x [**BC**]- 1.669x10^-6^ x [**A^2^**] - 1.107x10^-6^x [**B^2^**] + 1.043x10^-13^x [**C^2^**] | | |
| **IDM =** | -0.225+7.318x10^-3^x [**A**]+0.016x [**B**]-1.277x10^-6^x [**C**]-1.875x10^-4^x [**AB**]+2.000x10^-8^ x [**AC**]-1.125x10^-7^x [**BC**]-4.912x10^-4^x [**A^2^**]+1.600x10^-3^x [**B^2^**] +3.375x10^-12^x[**C^2^**] | -0.231+0.007x[**A**]+9.825x10^-3^x[**B**]-1.264x10^-6^x[**C**]-1.875x10^-4^x[**AB**]+2.000x10^-8^x [**AC**] - 1.125x10^-7^x [**BC**] -4.912x10^-4^x [**A^2^**] + 1.600x10^-3^x[**B^2^**]+ 3.375x10^-12^x[**C^2^**] | | |
| **Entropy =** | 21.939-0.325x[**A**]-0.664x[**B**]-3.550x10^-4^x[**C**]-1.875x10^-4^x[**AB**]+4.387x10^-7^x [**AC**]+2.812x10^-6^x[**BC**]+2.160x10^-3^x[**A^2^**]+0.181 x [**B^2^**]- 1.393x10^-10^x[**C^2^**] | 21.472-0.321x[**A**] - 0.489x[**B**] -4.418x10^-4^x[**C**]-1.875x10^-4^x[**AB**] +4.388x10^-7^x[**AC**] +2.812x10^-6^x [**BC**] + 2.160x10^-3^x[**A^2^**] +0.182x[**B^2^**] -1.393x10^-10^x[**C^2^**] | | |
